# Sex differences in amino acids lost via sweating could lead to differential susceptibilities to disturbances in nitrogen balance and collagen turnover

**DOI:** 10.1007/s00726-017-2431-4

**Published:** 2017-05-04

**Authors:** R. H. Dunstan, D. L. Sparkes, B. J. Dascombe, C. J. Stevens, G. R. Murphy, M. M. Macdonald, J. Gottfries, C.-G. Gottfries, T. K. Roberts

**Affiliations:** 1 University of Newcastle, Callaghan, NSW 2308 Australia; 2 Latrobe University, Melbourne, VIC 3086 Australia; 3 Southern Cross University, Coffs Harbour, NSW 2450 Australia; 4 University of Gothenburg, Box 100, 405 30 Gothenburg, Sweden; 5 Gottfries Clinic, Mölndal, Sweden

**Keywords:** Amino acid, Faux sweat, Urine, Protein turnover, Histidine, Chronic fatigue

## Abstract

Fluid collected during sweating is enriched with amino acids derived from the skin’s natural moisturising factors and has been termed “faux” sweat. Little is known about sex differences in sweat amino acid composition or whether faux sweat amino acid losses affect nitrogen balance. Faux sweat collected by healthy adults (*n* = 47) after exercise, and at rest by chronic fatigue patients, was analysed for amino acid composition. Healthy females had higher total amino acid concentrations in sweat (10.5 ± 1.2 mM) compared with healthy males (6.9 ± 0.9 mM). Females had higher levels of 13 amino acids in sweat including serine, alanine and glycine. Higher hydroxyproline and proline levels suggested greater collagen turnover in females. Modelling indicated that with conservative levels of exercise, amino acid losses in females via faux sweat were triple than those predicted for urine, whereas in males they were double. It was concluded that females were more susceptible to key amino acid loss during exercise and/or hot conditions. Females reporting chronic fatigue had higher levels of methionine in faux sweat than healthy females. Males reporting chronic fatigue had higher levels of numerous amino acids in faux sweat compared to healthy males. Higher amino acid loss in faux sweat associated with chronic fatigue could contribute to a hypometabolic state. Depending on activity levels, climatic conditions and gender, amino acid losses in sweat and skin leachate could influence daily protein turnover where periods of continuously high turnover could lead to a negative net nitrogen balance.

## Introduction

The primary role of sweating is thermoregulation which is achieved by evaporative cooling where the loss of fluid volume concomitantly facilitates excretion of electrolytes, urea, organic and amino acids (Kutyshenko et al. 2011). Most research of sweat has focussed on water and electrolyte losses. The amino acid composition of sweat is different to the plasma profile with several amino acids consistently reported at concentrations higher than those observed in the plasma including serine, histidine, ornithine, glycine, alanine, aspartic acid and lysine, while glutamine and proline have been reported at lower levels in sweat than in the plasma (Dunstan et al. 2016; Embden and Tachau 1910; Gitlitz et al. 1974; Hier et al. 1946; Kutyshenko et al. 2011; McSwiney 1934). The reduced levels of glutamine and proline suggested the presence of mechanisms for filtration or reabsorption of these amino acids (Dunstan et al. 2016; Liappis and Jakel 1975). The concentrations of amino acids in sweat can be highly variable between individuals (Liappis and Jakel 1975) but it was recently proposed that phenotypic subgroups exist with differential sweat volumes and amino acid composition which could explain much of the variance (Dunstan et al. 2016). Estimating amino acid losses via sweat is problematic as it must take into account contributions from the natural moisturising factors (NMF) in the skin, evaporative water losses, differential compositions of sweat from various skin locations and the logistics of sample collection (Dunstan et al. 2016; Rawlings and Matts 2005). Water is also lost via diffusion through the skin contributing to the “insensible water loss” pool (Dmitrieva and Burg 2011; Guyton and Hall 2000). Sweat collected within the first 30 min of exercise has initially higher levels of amino acids contributed from the skin surface where the process of wetting of the skin by sweat effectively leaches the free amino acids used as humectants in NMF (Dunstan et al. 2016). Thus, to evaluate nutrient loss via the process of sweating, the fluid collected has been referred to as “faux sweat” to represent the final nutrient and electrolyte composition in the fluid from the skin and sweat (Dunstan et al. 2016; Weschler 2008). If the plasma volume is assumed to be 3 L (Mosby’s Medical Dictionary 2009), then the quantity of amino acids in circulation was estimated to be around 6–7 mmol. Athletes can lose 1–2 L per hour of fluid through sweating with estimates of losses of amino acids at 5–23 mmol per hour of exercise in warm conditions (Dunstan et al. 2016). Such losses would place a considerable demand on protein turnover in the body to maintain homeostatic levels of amino acids in the plasma to support exercise while nutrients cannot be provided via ingestion (Dunstan et al. 2015, 2016).

It is well established that a net negative nitrogen balance can lead to poor health as demonstrated by studies where histidine deprivation led to a negative nitrogen balance and reduced haemoglobin levels (Clemens et al. 1984; Cooperman and Lopez 2002; Kopple and Swendseid 1975). Amino acids are also lost via urine and assessment of potential daily losses should be integrated with potential sweat losses for assessing net nitrogen balance. This is not easily achieved as 24-h urine samples contain contributions from dietary excesses and vary with fluid intake. In an attempt to address this issue, fasted first of the morning urine samples have been assessed to characterise homeostatic subtypes in the population and were also used to compare differences in urinary excretion of amino acids (Dunstan et al. 2017). Three population subgroups were constructed based on similarities in urinary excretion patterns from 151 healthy subjects (52 females and 99 males). It was noted that female urinary levels of both proline and hydroxyproline were strongly associated with reported pain intensity, while proline was associated with the symptom indices of fatigue, gastrointestinal function, sleep and vitality (Dunstan et al. 2017). Daily losses of nitrogen via faecal excretion also contribute to the net nitrogen balance representing around one-third of the quantity lost via urine (Bodwell et al. 1979; Tessari 2006). Nitrogen faecal excretion is normally relatively constant (Matthews 1999), but may vary under conditions of stress, trauma and disease.

It was proposed that healthy female adults experience higher rates of collagen turnover which would be more likely to result in a net negative nitrogen balance. Collagen is the most abundant structural protein in the body (Di Lullo et al. 2002; Kjaer and Hansen 2008) with various types located in connective tissues including cartilage, ligaments, tendons and skin as well as within the bones, blood vessels, gastrointestinal tract and muscle tissues. Collagen turnover is higher for women where, for example, turnover in human femoral mid-shaft collagen has been estimated to be around 4% per year for women from 20 years of age decreasing to 3% per year at 80 years, whilst males had a collagen turnover rate over the same ages of 3% decreasing to 1.5% (Hedges et al. 2007). It was also found that women have a lower rate of tendon collagen synthesis both in the resting state and following exercise suggesting that female sex hormones may influence collagen turnover (Hedges et al. 2007). It is, therefore, possible that collagen degradation occurs at a greater rate in females and that the rate of synthesis is slower in comparison to males. As collagen is ubiquitous throughout the body’s tissues, the presence of higher collagen turnover rates in females may provide a model for explaining the higher percentage of females showing susceptibility to a broad range of symptoms including persistent fatigue (Jason et al. 1999).

Therefore, the current study aimed to compare amino acid compositions of exercise-induced faux sweat in healthy adult males and females and those with chronic fatigue syndrome. It was proposed that healthy females would show higher levels of collagen amino acids proline, hydroxyproline, glycine, alanine, serine and aspartic acid in sweat in comparison to healthy males and that this would be exacerbated in association with chronic fatigue syndrome.

## Materials and methods

### Participants

The healthy participants were derived from two datasets. The first set was a group of 19 endurance athletes comprising 11 middle distance runners (5 km) and 8 triathletes who had sweat collected after performing self-paced time trials under a constant environment at 32–34 °C and 20–30% RH as described previously (Dunstan et al. 2016). A second set of participants were healthy female (*n* = 17) and male (*n* = 11) university students engaged in gym-based exercise classes providing a total of 47 healthy subjects. A smaller group of individuals who had indicated on a general health questionnaire that they had a diagnosis of chronic fatigue syndrome (CFS, 4 females and 3 males) were also recruited for the study. The age range of participants was 18–45 years. As part of a larger separate study, 151 healthy adults comprising 52 females and 99 males provided a fasted first of the morning urine sample for amino acid analysis (Dunstan et al. 2017). The urinary concentration data for the males and females were utilised in the current study for subsequent modelling of amino acid losses from urine and sweat under various defined scenarios. The research was approved by the University of Newcastle Human Ethics Committee (approval numbers: H-2010-1313, H-2014-0086, H-2011-0024 and H-2012-0311) and informed consent was obtained from all individual participants included in the study.

### Sample collection and amino acid analysis

Faux sweat was collected from the healthy cohort using sterile specimen jars (70 mL, Sarstedt, Germany) gently scraped over the skin surface of their forearm or back after 30–40 min of exercise. Faux sweat was collected from the fatigued cohort from their forearms which were enclosed in a plastic bag secured below the elbow while sitting at rest in a warm location. Sweat samples from the healthy individuals were stored at 4 °C and processed within 48 h of receipt. Sweat samples from the chronic fatigue patients were transferred to a Monovette^®^ 10-mL boric acid tube (Sarstedt, Germany) for transport.

Determination of the sample composition of a range of common amino acids and dipeptides was conducted by EZ:Faast™ derivatisation (esterification of amino acids) followed with analysis by gas chromatography with flame ionisation detection (FID) as described previously (Evans et al. 2008). EZ:Faast™ (Phenomenex^®^ Inc.) is a testing kit for GC analysis of physiological samples, suitable for detection and quantitation of over 40 aliphatic and aromatic amino acids and related compounds. The procedure consists of a solid phase extraction step followed by derivatisation and a liquid/liquid extraction in preparation for GC analysis. Urine and sweat sample volumes were 100 and 50 µL, respectively.

### Modelling losses from sweat and urine

The results for the amino acid concentrations in faux sweat from the current study (17 females, 30 males) and urine from an earlier study [52 females, average age ± SD of 35 ± 13.7; 99 males average age ± SD of 29.5 ± 11.8 (mean ± SD); Dunstan et al. 2017] were used to model potential losses against the context of the reservoir of amino acids maintained in 3 L of circulating plasma calculated from data in Armstrong and Stave (1973). Rates of fluid loss via urination and faux sweat vary greatly between individuals but for modelling purposes a daily urinary volume of 1500 mL was used (Koushanpour and Kriz 1986) and two levels of faux sweat losses were calculated at rates less than the maximum sweating rates for humans at 1–2 L per hour during exercise (Torii 1995). It was, therefore, deemed reasonable to present potential losses in sweat for comparison against urinary output and plasma loads under two scenarios where the daily volumes of sweat were 0.5 L (low activity, 24 °C) and 2 L (exercise, 24 °C).

### Statistical analysis

The sweat amino acid relative abundance data were arcsine-transformed to improve normality. The data from the CFS and healthy groups were combined and subjected to k-means clustering analysis to determine whether discrete groups based on sweat amino acid profiles were discernible. Amino acid concentration data from each of the groups generated by k-means clustering were compared using ANOVA and Tukey’s HSD test for unequal sample sizes. Principle component analysis was performed on arcsine-transformed amino acid data. All statistics were performed using Dell Statistica version 13 (Dell Inc. 2015). Results were considered statistically significant at *p* < 0.05.

## Results

Comparisons of the healthy 30 males and 17 females showed that 13 amino acids had significantly higher concentrations in faux sweat in females compared with the males resulting in a higher total concentration of amino acids in faux sweat from the females (Table 1). The levels of serine, alanine and glycine represented the most abundant components in faux sweat from the females and were significantly higher than the respective levels in faux sweat from the males. Many essential amino acids such as threonine, valine and tyrosine as well as non-essential proline and hydroxyproline were also significantly higher in female faux sweat (*p* < 0.05).

**Table 1:** A summary of significant differences in sweat amino acid concentrations between healthy females and males, and between females and males who reported chronic fatigue

Amino acid (µmol/L)	Concentration (SE)			
	Healthy females (*n* = 17)	Healthy males (*n* = 30)	CFS females (*n* = 4)	CFS males (*n* = 3)
Serine	2702 (360)^a^	1337 (156)	3393 (651)	3790 (865)^c^
Alanine	1396 (155)^a^	665 (92)	1712 (330)	1961 (555)^c^
Glycine	1704 (233)^a^	923 (117)	2310 (451)	2682 (611)^c^
Aspartic acid	585 (83)^a^	305 (46)	1216 (414)^b^	1055 (339)^c^
Threonine	534 (93)^a^	240 (28)	448 (182)	180 (180)
Glutamic acid	491 (80)^a^	256 (38)	303 (79)	359 (116)
Valine	396 (44)^a^	237 (30)	588 (162)	592 (179)^c^
Proline	272 (30)^a^	122 (16)	298 (137)	426 (118)^c^
Tyrosine	238 (27)^a^	108 (20)	350 (95)	345 (122)^c^
Asparagine	170 (25)^a^	86 (14)	325 (100)^b^	296 (121)^c^
Hydroxyproline	23 (4)^a^	6 (2)	39 (20)	41 (12)^c^
Cystine	2.8 (0.6)^a^	1.0 (0.4)	3.3 (1.4)	4.1 (2.1)^c^
Methionine	17.6 (3)^a^	6.8 (2.1)	40 (11)^b^	46 (15)^c^
Total	10,534 (1180)^a^	6940 (887)	14,223 (3106)	14,845 (4161)^c^

^a^Statistically significant difference (*p* < 0.05) between healthy females and healthy males

^b^Statistically significant difference between healthy females and chronic fatigue females

^c^Statistically significant difference between chronic fatigue males and healthy males

The amino acid compositions in faux sweat for subjects reporting chronic fatigue were then compared separately for females and males in Table 1. With low replicate numbers for the CFS group the variances were high but significant differences were apparent. The females with chronic fatigue had significantly higher levels of aspartic acid, asparagine, cystine and methionine (*p* < 0.05) compared with the healthy females. The males with chronic fatigue had significantly higher levels in serine, alanine, glycine, aspartic acid, valine, proline, tyrosine, asparagine, hydroxyproline, cystine and methionine compared with the healthy males (*p* < 0.05). The total mean level of amino acids in faux sweat for the males with chronic fatigue was significantly higher than the healthy males and similar to the mean level measured for the females with chronic fatigue.

**Fig. 1:**
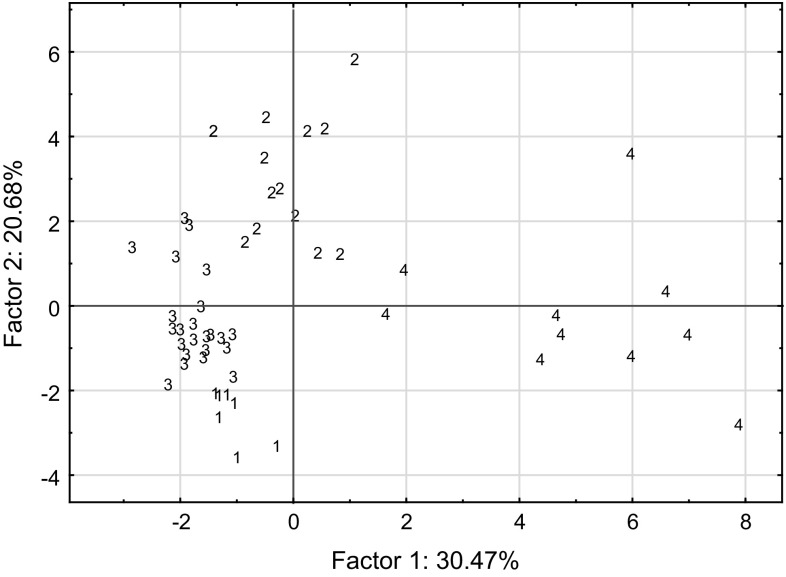
Principle component analysis (PCA) of the relative (%) abundance of amino acids in sweat: the scatterplot plot of the PCA scores for factor 1 vs factor 2 where each case was coded for membership of one of the four clusters (1–4) defined by k-means clustering

The amino acid concentration data were converted to relative (percentage) abundances for analysis by k-means clustering. The results revealed that four clusters could be differentiated with minimum group membership set at *n* > 6, and the relative abundance as well as the mean concentration data for these clusters have been summarised in Table 2. Most of the healthy females were assigned to cluster 3, while most of the healthy males were evenly distributed between clusters 2, 3 and 4. It was noted that all 19 endurance athletes were assigned into either cluster 2 or 4, where 7 (out of 8) triathletes formed part of cluster 2 and 8 (out of 11) middle distance runners were in cluster 4. The remaining healthy males were primarily assigned to cluster 3. The amino acid composition of each cluster was dominated by four major components which together comprised 57–61% of the amino acids. Serine, glycine, alanine and histidine/aspartic acid were the predominant amino acids in the faux sweat profiles for clusters 1, 2 and 3, and histidine, serine, ornithine and glycine were the most abundant for cluster 4 (Table 2). Cluster 1 was distinguished by having the highest concentrations of amino acids in sweat which included significantly higher concentrations of certain essential amino acids compared with other clusters including methionine, phenylalanine, tyrosine and tryptophan (*p* < 0.05). In clusters 1 and 3 hydroxyproline was present in the sweat whereas there was an absence of this amino acid in the faux sweat of clusters 2 and 4. Participants who reported a diagnosis of CFS with persistent fatigue for more than 3 years were found to be allocated to either cluster 1 or 3 on the basis of their amino acid sweat profiles. The all-male cluster 4 was characterised by having the highest concentrations of histidine, lysine and ornithine of any of the groups. The relative abundance data for the amino acids were subjected to principle component analysis (PCA) and the results are presented in Fig. 1 where individual cases were colour coded based upon cluster membership as determined by k-means clustering. It was clear from the scatterplot that the cases from each cluster were well resolved from each other. The cases in cluster 4 were spread along factor 1 which was aligned with the contributions from histidine, ornithine and lysine as determined by the factor loadings.

**Table 2:** Characteristics of four clusters generated by k-means clustering based on relative abundances of amino acids in sweat for both healthy and CFS participants

	Cluster 1 (*n* = 7)		Cluster 2 (*n* = 14)		Cluster 3 (*n* = 23)		Cluster 4 (*n* = 10)		Cluster differences (*p* < 0.05)
Number									
Females	2		3		12		0		
Males	1		11		8		10		
CF females	2		0		2		0		
CF males	2		0		1		0		
Most abundant sweat amino acids expressed as percentage (%) of all amino acids									
	Serine	23.5	Serine	29.1	Serine	24.5	Histidine	19.7	
	Glycine	14.1	Glycine	14.2	Glycine	17.4	Serine	13.8	
	Alanine	13.1	Alanine	9.7	Alanine	12.7	Ornithine	12.0	
	Aspartate	7.6	Histidine	7.1	Aspartate	6.1	Glycine	11.5	
	Total	58.3	Total	60.1	Total	60.7	Total	57.1	
BCAA in sweat, µmol/L, (SE)									
Leucine	489 (61)		122 (24)		227 (23)		341 (54)		C1 > C2 = C3; C4 > C2
Isoleucine	350 (37)		83 (18)		167 (17)		184 (34)		C1 > C2 = C3; C1 > C4 = C3; C4 > C2
Valine	690 (69)		173 (36)		348 (37)		274 (47)		C1 > C3 > C2; C1 > C4 = C3; C4 = C2
Total	1529		378		742		799		C1 > C2 = C3; C4 > C2
Essential amino acids (non-BCAA), µmol/L, (SE)									
Cystathionine	5.1 (1.2)		0		1.7 (0.8)		0		C1 = C3 > C2 = C4
Cystine	6.7 (0.6)		0		2.4 (0.4)		0		C1 > C3 > C2 = C4
Histidine	886 (88)		344 (74)		525 (60)		2024 (527)		C4 > C1 = C2 = C3
Lysine	404 (46)		112 (27)		183 (19)		877 (202)		C4 > C1 = C2 = C3
Methionine	45 (7)		2 (1)		19 (2)		1 (1)		C1 > C3 > C2 = C4
Phenylalanine	297 (35)		65 (13)		129 (13)		197 (32)		C1 > C3 = C2; C4 > C2
Threonine	263 (132)		369 (120)		443 (33)		139 (28)		ns
Tryptophan	128 (18)		20 (7)		70 (8)		137 (33)		C1 > C2; C4 > C3 = C2
Tyrosine	397 (50)		114 (36)		199 (18)		77 (38)		C1 > C2 = C3 = C4
Non-essential amino acids, µmol/L, (SE)									
Alanine	2002 (203)		528 (152)		1208 (129)		724 (134)		C1 > C2 = C4; C3 > C2
Asparagine	357 (54)		53 (13)		163 (20)		66 (10)		C1 > C3 > C2; C1 > C4
Aspartic acid	1218 (236)		270 (55)		560 (66)		192 (38)		C1 > C2 = C3 = C4
Glutamic acid	537 (68)		250 (88)		384 (55)		222 932)		ns
Glutamine	78 (12)		83 (18)		106 (26)		73 (34)		ns
Glycine	2298 (469)		765 (226)		1616 (159)		999 (177)		C1 > C2 = C4; C3 > C2
Hydroxylysine	67 (8)		10 (8)		50 (7)		82 (28)		C4 > C2
Hydroxyproline	45 (4)		0		23 (3)		0		C1 > C3 > C2 = C4
Ornithine	909 (202)		212 (56)		325 (38)		1255 (329)		C4 > C2 = C3; C4 > C3
Proline	375 (86)		119 (28)		242 (24)		89 (11)		C1 > C2 = C4; C3 > C4
Serine	3647 (442)		1700 (504)		2189 (185)		1219 (226)		C1 > C2 = C4; C1 > C4
Total amino acids in sweat, µmol/L, (SE)									
	15,573 (3235)		5414 (1434)		9188 (862)		9258 (1761)		C1 > C2

To evaluate differences between males and females in potential losses of amino acids, a loss of 0.5 L of faux sweat per day was taken to represent a scenario without strenuous exercise at a standard temperature of 24 °C. A loss of 2 L per day was proposed to represent a day with a reasonable level of exercise activity at a standard temperature of 24 °C. Comparisons of the urine and sweat compositions from the two study groups revealed that histidine and glycine were the most highly abundant amino acids measured in both sweat and urine, where sweat also contained very high levels of serine in both males and females (Table 3). The total amino acid levels in urine did not vary significantly between males and females (Dunstan et al. 2017) but the total level of amino acids in the faux sweat from females was significantly higher than that found in the males (*p* < 0.05). Under the scenario of a sedentary day with minimal exercise and no exposure to heat, the losses via faux sweat in males and females were lower than the estimated daily output from urine. In scenario 2 when the sweat rate was elevated to 2 L to account for inclusion of exercise in the day, the model for total losses of amino acids was nearly double the level in urine for males and triple the level in urine for females. In males, 78% of the total loading of the amino acids circulating in plasma is estimated to be lost for every litre of sweat, and in females 116% of the plasma load is estimated to be lost in one litre of sweat.

**Table 3:** Comparison of urinary, sweat and plasma amino acids with calculated losses based on estimated potential average daily urinary and sweat excretion rates

Amino acid	Urine^a^	Sweat	Total in 3 L^b^ plasma	Total^a^ in 1.5 L urine	Scenario 1 calculated total in 0.5 L faux sweat	Scenario 2 calculated total in 2 L faux sweat
Mean µmoles/L	Male/female					
Essential						
Histidine	1315/1041	944/653	267	1973/1562	472/327	1888/1306
Lysine	263/234	387/205	594	395/351	194/103	774/410
Branched-chain						
Leucine	32/29	235/250	480	48/44	118/125	470/500
Isoleucine	10/11	145/188	252	15/17	73/94	290/376
Valine	45/45	259/396^a^	756	68/68	130/198	518/792
Non-essential						
Glycine	975/1198	923/1704^a^	708	1463/1797	462/852	1846/3408
Proline	8/11	122/272^a^	717	11/16	61/136	244/544
Alanine	264/251	665/1396^a^	1,257	396/337	333/698	1330/2792
Serine	315/360	1367/2702^a^	342	473/540	684/1351	2734/5404
Aspartic acid	14/21	305/585^a^	21	21/32	153/293	610/1170
Glutamine	542/476	78/95	1935	813/714	39/48	156/190
Total^c^	5185/4955	6940/10,534^d^	8886	7778/7433	3470/5267	13,880/21,068

^a^Dataset derived from Dunstan et al. (2017) where only the male and female total values were reported

^b^Calculated from data in Armstrong and Stave (1973)

^c^“Total” values include the evaluations of amino acids in addition to those listed in the table)

^d^Significantly different between males and females (*p* < 0.05)

## Discussion

The total concentration of amino acids in faux sweat collected during exercise from healthy adult females was higher than that measured in healthy adult males. The higher levels were primarily due to increased concentrations of serine, alanine and glycine for the females and were consistent with the results of an earlier study which reported comparatively higher amino acid levels in sweat for females (Liappis and Jakel 1975). The average levels of several amino acids including serine, glutamic acid, histidine and glycine were many times more concentrated in the faux sweat for females in the current study compared with the literature values for plasma (Armstrong and Stave 1973). To a lesser extent, the males mirrored similar higher concentrations in the faux sweat relative to plasma. The higher individual and total amino acid concentrations seen for the female cohort could be reflective of gender differences in sweating efficiencies which include variations in the production of NMF in the stratum corneum. Sweat fluid losses have been shown to be higher for men (Mehnert et al. 2002) which, in the current study, could have resulted in comparatively more dilute sweat with regards to amino acid concentration for the males. The results suggested that sweat composition may also be associated with levels of fitness and the type of sport where there appeared to be differences between the two types of male endurance athletes and males undergoing gym workouts in this study.

The development of a model to compare amino acid excretion losses in males and females involved a number of assumptions but provided insight for understanding differences between males and females in regard to nitrogen balance. The urine excretion data derived from overnight fasted samples were taken to reflect metabolic homeostasis with minimal contributions from dietary excesses. Protein turnover would be higher during the waking hours to support exercise, recovery and repair processes and it was proposed that the early morning measure would provide a conservative estimate of likely urine excretion losses of amino acids when combined with an average urinary output. It was acknowledged that urinary excretion and losses via faux sweat would vary depending on temperature, humidity and activity levels, as well as underlying genetic factors regulating body characteristics such as body composition and fitness level. The development of the model to compare losses of amino acids was based on findings that training and competition in most sports at temperatures ranging from 19 to 33 °C generate sweating rates of 1–2 L per hour for males and females (Rehrer and Burke 1996; Torii 1995). Second, studies reported that workers in prolonged hot conditions could lose 10–12 L of sweat fluid per day (Bates and Miller 2008; Jessen 2000; Mack and Nadel 1996; Sawka et al. 1996). The current model demonstrated that females were more susceptible to amino acid losses during exercise and/or exposures to hot conditions. These comparisons also demonstrated that not all amino acids were lost equally in urine and sweat. The process of kidney reabsorption was very efficient for the branched-chain amino acids and proline but histidine and glycine were lost in urine at more than four times the concentrations measured in the plasma. In the sweat, there were substantial losses of serine, alanine, glycine, histidine and aspartic acid. This represents a considerable demand on plasma resources of amino acids which must be maintained as a constant reservoir to service the requirements of the body.

To ensure amino acid homeostasis in the plasma whilst food ingestion is not possible during exercise, amino acids are derived via proteolysis of non-myofibrillar proteins to provide amino acids for energy, recovery and repair (Phillips 2004; Poortmans et al. 2012). The requirements for new protein synthesis can remain elevated after exercise for 24–36 h in athletes and up to 48 h in untrained individuals (Phillips et al. 1999). It was, therefore, hypothesised that females may be more susceptible to developing a net negative nitrogen balance as a result of high-intensity exercise, exposure to hot climatic conditions, poor diet, stress, injury, pathogenic challenge or various combinations of these scenarios. It was acknowledged that to strengthen the model, further research is required to provide better estimates of daily urinary and sweat losses. However, the modelling does suggest that once physical activity (or ambient temperature) is increased with concomitant increases in faux sweat production, the potential for amino acid loss via sweat far exceeds losses from urine. An individual’s level of physical activity and operating environment should be considered in developing strategies for optimising nutrient intake to maintain nitrogen balance. While athletes are recommended to increase protein intake following resistance exercise and high-intensity exercise for muscle repair (Phillips and VanLoon 2011), no recommendations currently exist in regard to increasing amino acid intake during or immediately after exercise in the heat to minimise catabolism and maintain nitrogen balance.

The losses of glycine, proline, alanine and serine via faux sweat may limit muscle maintenance, repair and recovery processes associated with exercise. Glycine is the most abundant collagen amino acid representing approximately 33% of the amino acid content of the protein, while proline comprises 22% of pro-collagen with almost half of the proline subsequently modified post-transcription to form hydroxyproline. Alanine comprises 11% and serine around 3.6% of the amino acid composition of collagen. Collagen proteins represent the largest family of proteins in the body (Di Lullo et al. 2002; Kjaer and Hansen 2008) and collagen is a major component of the endomysium which is the connective tissue surrounding the muscle fibres (Light and Champion 1984). Losses of these amino acids could potentially negatively impact collagen synthesis, particularly in females where the losses of proline in sweat were double than that of the males. This proposal was supported by the detection of higher levels of the collagen breakdown product hydroxyproline in the females. Losses of the key precursor amino acids for collagen synthesis may lead to amino acids such as glycine and proline becoming conditionally essential. The turnover of connective tissue may represent a key source of protein catabolism to support exercise which would be consistent with the high levels of collagen amino acids found in the female sweat following exercise.

Glycine and histidine were the amino acids found in the greatest concentrations in both sweat and urine which was consistent with earlier studies (Derezinski et al. 2017; Tan and Bajra 2006). Taurine was not measured in the current study but was also noted as being a highly abundant component of the urine as well as 3-methyl-histidine, which can be an indicator of muscle protein turnover (Derezinski et al. 2017). The high sweat concentrations in combination with high urinary losses of histidine suggested that increases in exercise and exposures to high temperatures would place additional demands on the intake of histidine. Histidine is important in haemoglobin production and depletion can lead to anaemia and associated fatigue that may be corrected by provision of dietary histidine (Clemens et al. 1984; Cooperman and Lopez 2002; Kopple and Swendseid 1975). Sweat levels of histidine, glycine, serine and alanine were the key components differentiating the relative abundance profiles between the four clusters generated by k-means clustering techniques. In a previous study, urinary concentrations of histidine, glycine, serine and alanine were also major factors discriminating between three clusters (Dunstan et al. 2017). The body’s capacity to retain these key amino acids by minimising losses occurring via urine and sweat (including amino acids leached from the skin surface) would be important in maintaining nitrogen balance (Phillips and VanLoon 2011). Such an association would compound an individual’s susceptibility to amino acid depletion potentially resulting in a negative nitrogen balance under conditions of increased exercise, insufficient protein intake, pathogenic challenge, injury, stress or trauma.

Comparisons of the females reporting chronic fatigue with the healthy females revealed increased losses in faux sweat of the sulphur-containing amino acid methionine. Further losses of asparagine and aspartic acid were also noted. The males reporting CFS had high variance but the values reflected a substantial upward shift in the amount of amino acids lost via the faux sweat with nearly all amino acids showing significant increases compared to the healthy males. In addition to glycine and alanine, the males with fatigue also had significantly higher levels of proline and hydroxyproline, indicative of higher rates of collagen turnover compared with the healthy males. The patients with chronic fatigue were not asked to undergo exercise to generate sweat and the passive mode of collection could result in some differences between the faux sweat compositions. However, females with CFS had higher total concentrations of amino acids in the faux sweat compared with the healthy females (not statistically significant) but the CFS males had more than double the concentration of amino acids compared with the healthy males. These results suggested that amino acid losses via the skin through insensible water loss, leaching of NMF and sweating in patients with chronic fatigue could contribute to a net negative balance with impact on fatigue and metabolic homeostasis. These outcomes were consistent with a recent report of altered metabolic homeostasis in patients with chronic fatigue which determined that 80% of measured metabolites were decreased in patients with CFS indicative of a hypometabolic state (Naviaux et al. 2016). The broad range of tissues, organs and functions which could be affected by impaired collagen turnover and the loss of key collagen amino acids by women suggested a new avenue of research regarding the aetiology of CFS.

## Conclusion

The process of sweat-facilitated losses of amino acids represents a significant pathway for loss of key amino acids with exercise and exposure to warmer conditions. Females displayed higher concentrations of amino acids in faux sweat compared with males. Glycine and histidine were the major components lost in both sweat and urine and thus, under high-intensity exercise regimes and conditions of high sweat volume, these might represent potential limiting factors influencing rates of protein turnover to support exercise metabolism, repair and recovery processes. Higher levels of proline, hydroxyproline, glycine, alanine, serine and aspartic acid in sweat from females were consistent with higher rates of collagen turnover in females. The male subjects reporting a diagnosis of CFS showed a higher capacity to lose amino acids via faux sweat than healthy males. It was concluded that losses of amino acids in sweat could significantly contribute to the status of nitrogen balance in humans.

## References

[CR1] Armstrong MD, Stave U (1973). A study of plasma free amino acid levels. II. Normal values for children and adults. Metabolism.

[CR2] Bates GP, Miller VS (2008). Sweat rate and sodium loss during work in the heat. J Occup Med Toxicol.

[CR3] Bodwell CE, Schuster EM, Kyle E, Brooks B, Womack M, Steele P, Ahrens R (1979). Obligatory urinary and fecal nitrogen losses in young women, older men, and young men and the factorial estimation of adult human protein requirements. Am J Clin Nutr.

[CR4] Clemens RA, Kopple JD, Swendseid ME (1984). Metabolic effects of histidine-deficient diets fed to growing rats by gastric tube. J Nutr.

[CR5] Cooperman JM, Lopez R (2002). The role of histidine in the anemia of folate deficiency. Exp Biol Med.

[CR6] Derezinski P, Klupczynska A, Sawicki W, Palka JA, Kokot ZJ (2017). Amino acid profiles of serum and urine in search for prostate cancer biomarkers: a pilot study. Int J Med Sci.

[CR7] Di Lullo GA, Korkko SM, Ala-Kokko L, San Antonio JD (2002). Mapping the ligand-binding sites and disease-associated mutations on the most abundant protein in the human, Type I collagen. J Biol Chem.

[CR8] Dmitrieva NI, Burg MB (2011). Increased insensible water loss contributes to aging related dehydration. PLoS One.

[CR9] Dunstan RH (2015). Sweat facilitated losses of amino acids in Standardbred horses and the application of supplementation strategies to maintain condition during training. Comp Exerc Physiol.

[CR10] Dunstan RH (2016). Sweat facilitated amino acid losses in male athletes during exercise at 32–34 °C. PLoS One.

[CR11] Dunstan RH (2017). Diverse characteristics of the urinary excretion of amino acids in humans and the use of amino acid supplementation to reduce fatigue and sub-health in adults. Nutr J.

[CR12] Embden G, Tachau H (1910). The occurrence of serine in human sweat. (abstract). Biochemische Zeitschrift.

[CR13] Evans C (2008). Altered amino acid excretion in children with autism. Nutr Neurosci.

[CR14] Gitlitz PH, Sunderman FW, Hohnadel DC (1974). Ion-exchange chromatography of amino acids in sweat collected from healthy subjects during sauna bathing. Clin Chem.

[CR15] Guyton AC, Hall JE, Guyton AC, Hall JE (2000). The body fluids compartments: extracellular and intracellular fluids: interstitial fluids and edema. Textbook of medical physiology.

[CR16] Hedges REM, Clement JG, Thomas DL, O’Connell TC (2007). Collagen turnover in the adult femoral mid-shaft: modeled from anthropogenic radiocarbon tracer measurements. Am J Phys Anthropol.

[CR17] Hier SW, Cornbleet T, Bergeim O (1946). The amino acids of human sweat. J Biol Chem.

[CR18] Jason LA (1999). A community-based study of chronic fatigue syndrome. Arch Intern Med.

[CR19] Jessen C (2000). Temperature regulation in humans and other mammals.

[CR20] Kjaer M, Hansen M (2008). The mystery of female connective tissue. J Appl Physiol.

[CR21] Kopple JD, Swendseid ME (1975). Evidence that histidine is an essential amino acid in normal and chronically uremic man. J Clin Investig.

[CR22] Koushanpour E, Kriz W (1986) Body fluids: turnover rates and dynamics of fluid shifts. In: Renal physiology, Principles, structure and function, 2nd edn. Springer, New York, pp 21–40

[CR23] Kutyshenko VP, Molchanov M, Beskaravayny P, Uversky VN, Timchenko MA (2011). Analyzing and mapping sweat metabolomics by high-resolution NMR spectroscopy. PLoS One.

[CR24] Liappis N, Jakel A (1975). Free amino acids in human eccrine sweat. Arch Dermatol Res.

[CR25] Light N, Champion AE (1984). Characterization of muscle epimysium, perimysium and endomysium collagens. Biochem J.

[CR26] Mack GW, Nadel ER, Fregly MJ, Blatteis CM (1996). Body fluid balance during heat stress in humans. Handbook of Physiology. Section 4: environmental physiology.

[CR27] Matthews DE, Shils ME, Olson JA, Shike M, Ross AC (1999). Proteins and amino acids. Modern nutrition in health and disease.

[CR28] McSwiney B (1934). The composition of human perspiration (Samuel Hyde Memorial Lecture): (Section of Physical Medicine). Proc R Soc Med.

[CR29] Mehnert P, Brode P, Griefahn B (2002). Gender-related difference in sweat loss and its impact on exposure limits to heat stress. Int J Ind Ergon.

[CR30] Mosby’s Medical Dictionary (2009) Plasma volume [Def1]. Farlex. http://medical-dictionary.thefreedictionary.com/plasma+volume. Accessed Feb 8 2017

[CR31] Naviaux RK (2016). Metabolic features of chronic fatigue syndrome. PNAS.

[CR32] Phillips SM (2004). Protein requirements and supplementation in strength sports. Nutrition.

[CR33] Phillips SM, VanLoon LJC (2011). Dietary protein for athletes: from requirements to optimum adaptation. J Sports Sci.

[CR34] Phillips SM, Tipton KD, Ferrando AA, Wolfe RR (1999). Resistance training reduces the acute exercise-induced increase in muscle protein turnover. Am J Physiol.

[CR35] Poortmans JR, Carpentier LO, Pereira-Lancha LO, Lancha A (2012). Protein turnover, amino acid requirements and recommendations for athletes and active populations. Braz J Med Biol Res.

[CR36] Rawlings AV, Matts PJ (2005). Stratum corneum moisturization at the molecular level: an update in relation to the dry skin cycle. J Invest Dermatol.

[CR37] Rehrer NJ, Burke LM (1996). Sweat losses during various sports. Austr J Nutr Diet.

[CR38] Sawka ML, Wenger CB, Pandolf KB, Fregly MJ, Blatteis CM (1996). Thermoregulatory responses to acute exercise-heat stress and heat acclimation. Handbook of Physiology. Section 4: environmental physiology.

[CR39] Tan I-K, Bajra B (2006). Plasma and urine amino acid profiles in a healthy adult population of Singapore. Ann Acad Med Singap.

[CR40] Tessari P, Mantovani G (2006). Nitrogen balance and protein requirements: definition and measurements. Cachexia and wasting: a modern approach.

[CR41] Torii M (1995). Maximal sweating rate in humans. J Hum Ergol.

[CR42] Weschler LB (2008). Sweat electrolyte concentrations obtained from within occlusive coverings are falsely high because sweat itself leaches skin electrolytes. J Appl Physiol.

